# Overcoming Poor Solubility of Dimenhydrinate: Development, Optimization and Evaluation of Fast Dissolving Oral Film

**DOI:** 10.15171/apb.2018.081

**Published:** 2018-11-29

**Authors:** Yuvraj Govindrao Jadhav, Upendra Chandrakant Galgatte, Pravin Digambar Chaudhari

**Affiliations:** Department of Pharmaceutics, Modern College of Pharmacy, Sector 21, Yamunanagar, Nigdi, Pune 411 044, Maharashtra, India.

**Keywords:** Antiemetic, Dimenhydrinate, Fast dissolving film, HPMC E5, Superdisintegrants

## Abstract

***Purpose:*** To develop fast dissolving oral film to address vomiting and nausea in pediatric population.

***Methods:*** Oral films of Dimenhydrinate were prepared by solvent casting method by using hydroxypropylmethyl cellulose E5 (HPMC E5), polyethylene glycol 400 (PEG 400) and croscarmellose sodium. Solubility of dimenhydrinate was enhanced by ethanol as a co-solvent. To make dimenhydrinate palatable sodium saccharin and peppermint oil were used. All films were evaluated for mechanical parameters, surface pH, morphology, disintegration time and percent dissolution.

***Results:*** Films were smooth, acceptable and white in colour. For optimized batch, drug content (99.106%), disintegration time (25 sec), dissolution (99.10% in 210 sec), surface pH (6.81) were acceptable.

***Conclusion:*** Optimized batch, due to its potential to deliver through fast dissolving film, can be developed for clinical use.

## Introduction

Fast dissolving oral film disintegrates or dissolves in oral cavity without need of water. This is the best alternative to tablets.^1^ Fast dissolving oral film is designed as a very thin, and instantly wetting by saliva if put on a tongue. Subsequently it disintegrates or dissolves once hydrated and release medicaments immediately. It gives fast absorption and thereby early bioavailability of drugs due to high surface area, high blood flow and permeability to pre-gastric mucosa.^2^ Oral film facilitates drug administration to paediatric, old age and non- cooperating patients.^3^ For better bioavailability, improvisation in solubility of drug is needed.^4^ Pre-gastric absorption avoids first pass metabolism.^5^ This ultimately results in increase in bioavailability compared to other dosage forms.^6^

A large population is affected by the vomiting and nausea. Nausea is the subjective and patient reports an unpleasant sensation in throat, diaphoresis, dizziness and excess salivation followed by vomiting. In vomiting contraction of abdominal muscles carryout the actual expulsion of stomach contents through the mouth. We have to control this act of vomiting in the emetic centre in the brain. Dimenhydrinate is a drug of choice for this study. Its overall antiemetic property is due to its effect as antihistaminic and anti cholinergic. Chemically, dimenhydrinate is a salt of diphenhydramine and 8-chlorotheophylline. Diphenhydramine is an antihistaminic and antagonistic at the H_1_ receptor and 8-chlorotheophylline is used to counteract drowsiness of diphenhydramine.

The present work was aimed to develop fast dissolving oral film of poorly water soluble drug, dimenhydrinate; using water soluble polymer.

## Materials and Methods

### Materials 

Dimenhydrinate (drug) was a gift sample from S. S. Pharmachem, Mumbai, India. HPMC E5 (film forming polymer), PEG 400 (plasticizer), citric acid (saliva stimulating agent) and sodium saccharin (sweetener) were procured from Loba Chemie, Mumbai, India. Croscarmellose sodium (superdisintegrant) was obtained from Research Lab. The remaining ingredients were of analytical grade.

### Methods 

#### Development of fast dissolving oral film of dimenhydrinate 

Films were developed by solvent casting method. HPMC E5 was dissolved in 5mL distilled water while stirring on magnetic stirrer. Drug was dissolved in 3mL distilled water and 3mL ethanol i.e. 1:1 solvent mixture. Ethanol was used as cosolvent to enhance solubility of drug in water. After keeping for half an hour; PEG 400, croscarmellose sodium, saccharin sodium, citric acid, peppermint oil were dissolved one by one in polymer solution with stirring on magnetic stirrer. Then, drug solution was added in a polymer solution and again stirred until complete mixing take place. Then, solution was casted on petri-plate and kept for drying at room temperature for next 24 hours. After drying, film was removed from petri-plate and cut into desired size (1.5cm x 3cm) containing 15 mg dose of drug.^7,8^

#### Optimization by 3^2^ factorial design 

According to 3^2^ full factorial design^9^ possible combinations are mentioned in Table 1. The amount of HPMC E5 and PEG 400 were independent variables. Percent drug release and disintegration times were dependent variables. Two independent factors, the concentrations of HPMC E5 and PEG 400 were chosen at low (-1), middle (0) and high (+1) levels. Concentration of HPMC E5 at low level was 300 mg, middle level 400 mg and at high level 500 mg. Concentration of PEG 400 at low level was 0.3 ml, middle level 0.4 ml and at high level 0.5ml. Design Expert version 8.0.7.1 software was used for optimization and validation of batches.

**Table 1:** Compositions of factorial batches of dimenhydrinate fast dissolving oral film

**Sr. No.**	**Components(mg)**	**F1**	**F2**	**F3**	**F4**	**F5**	**F6**	**F7**	**F8**	**F9**
**1**	Dimenhydrinate (mg)*	261.7	261.7	261.7	261.7	261.7	261.7	261.7	261.7	261.7
**2**	HPMC E5 (mg)	300	400	500	300	400	500	300	400	500
**3**	PEG 400 (mL)	0.3	0.3	0.3	0.4	0.4	0.4	0.5	0.5	0.5
**4**	Croscarmellose sodium (mg)	15	15	15	15	15	15	15	15	15
**5**	Saccharin sodium (mg)	80	80	80	80	80	80	80	80	80
**6**	Citric acid (mg)	80	80	80	80	80	80	80	80	80
**7**	Peppermint oil (mL)	0.2	0.2	0.2	0.2	0.2	0.2	0.2	0.2	0.2
**8**	Ethanol (mL)	3	3	3	3	3	3	3	3	3
**9**	Distilled Water (mL)	8	8	8	8	8	8	8	8	8

* Quantity was calculated based on area of petri- plate.

#### Evaluation 

Weights of films were taken in triplicate by electronic balance and average weight was calculated. Folding endurance was measured manually at an angle of 180° for all films. A film was folded repeatedly at the same place till it ruined. Folding endurance is the number of times the film could be folded without breaking.^10^ Drug content was determined by UV-Spectrophotometric method for all the batches. For this, 1.5 x 3 cm^2^ film was cut and dissolved in 100mL of phosphate buffer pH 6.8 and stirred it for an hour. The absorbance of the filtered solution was recorded at 278 nm and further drug content was calculated.^11^

In-vitro disintegration time was determined by putting a sample of a film in a glass beaker containing 25 mL distilled water and swirling till it broke. The disintegration time is one when film starts to rupture.^4,12^ Dissolution was run out by using USP type II apparatus using 900 mL phosphate buffer pH 6.8 as a medium at a paddle speed of 50 rpm. Samples of 5 mL were withdrawn periodically and the sample was replaced by same volume of fresh dissolution medium. The samples were assayed for drug content at 278 nm using UV-spectrophotometer.^13,14^

Surface pH was determined by putting a few drops of water on the film. The electrode was kept in intimate contact of a film and the pH was measured^7^. Scanning electron microscope (SEM; JEOL JSM- 6360, Japan) was used to observe the surface morphology of dimenhydrinate oral film.^15^

## Results and Discussion

### Development of fast dissolving oral film of dimenhydrinate 

Dimenhydrinate is poorly water soluble. However, co-solvent system improves solubility of water insoluble drug by changing polarity of solvent. So, ethanol (90% v/v) was used as a co-solvent in formulation of dimenhydrinate oral film. During drying, ethanol evaporates leaving behind no significant concentration in the film.

### Evaluation and Optimization of films

Films were smooth, non- transparent, acceptable and white in colour with good integrity.

Thin film shows quick disintegration and dissolution. While, thick film takes more time to disintegrate or dissolve. Thickness was measured by using calibrated Vernier calliper and showed thickness in the range of 0.151- 0.175 mm. Weights were shown to be minimum within the range of 70- 76 mg. The average weight of film was 73 mg. Uniformity of weight test was carried out as per Indian Pharmacopoeia 2007. Folding endurance showed strength and flexibility of film. Folding endurance depends on plasticizer concentration. Folding endurances of all factorial batches are shown in Table 2. This data revealed that films were having good mechanical strength with flexibility. Surface pH of a film and pH of an oral cavity (pH 6.8) should be closer. Formulation with pH 6.8 would get dissolved quickly in saliva and would be compatible with it. All factorial batches have shown pH in the range of 6.67 to 6.81 which is closer to 6.8 as shown in Table 2. Hence, it would not produce any irritation in a mouth.

The basic requisite of oral film dosage forms is its short disintegration time in the saliva. All factorial batches have shown disintegration time less than 60 sec. It is concluded that disintegration time increases with increase in polymer concentration due to viscosity of polymer and possibly due to formation of any cross linkage by the polymer with other excipients due to its hydrophilic nature. Parul et al.^13^concluded that disintegration time limit is 90 sec or less for the films obtained by them. On the other hand, Jain and Mundada^16^ have reported disintegration time for oral film 19 sec. However, there is no guidance officially regarding disintegration time for fast dissolving films. Considering the limits reported by these researchers, we found that our films were within the limits of acceptance (25 sec to 55 sec) of disintegration time.

It is a challenging task to get a desired drug content in a film. Factorial batches have shown satisfactory results of drug content and are shown in Table 2.

**Table 2:** Evaluation of fast dissolving oral film of dimenhydrinate

**Formulations**	**Folding endurance (n = 3)**	**Surface pH (n = 3)**	**Disintegration time (sec) (n = 3)**	**Percent drug content (n = 3)**
F1	24.00 ± 1.000	6.67 ± 0.05	28.66 ± 1.154	94.53 ± 0.597
F2	26.33 ± 1.527	6.77 ± 0.02	35.00 ± 3.000	91.98 ± 0.470
F3	36.33 ± 3.055	6.71 ± 0.01	54.00 ± 1.732	94.99 ± 0.544
F4	92.33 ± 4.509	6.74 ± 0.01	36.66 ± 1.527	95.98 ± 0.277
F5	44.33 ± 2.516	6.77 ± 0.05	46.33 ± 1.527	97.95 ± 0.493
F6	24.33 ± 3.214	6.77 ± 0.02	36.00 ± 1.732	96.25 ± 0.615
F7	26.66 ± 2.516	6.81 ± 0.03	25.00 ± 3.000	99.10 ± 0.745
F8	18.66 ± 2.081	6.78 ± 0.04	42.33 ± 2.519	94.22 ± 0.594
F9	18.66 ± 2.081	6.77 ± 0.06	45.66 ± 4.041	91.14 ± 0.649

**Figure 1:**
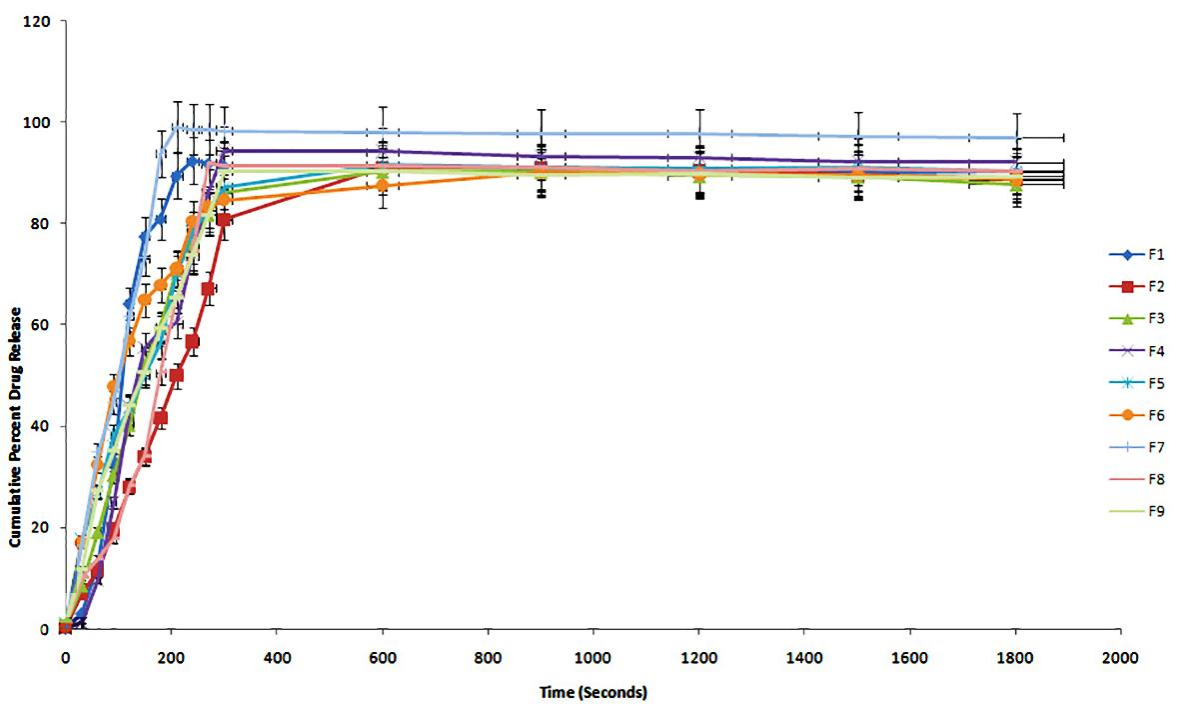
Comparative in- vitro dissolution study of all factorial batches in phosphate buffer pH 6.8

Dissolution study has shown that dissolution rate decreases with increase in polymer concentration and decrease in plasticizer concentration. The possible reason is high polymer concentration results in closer contacts of polymer particles which ultimately turn into formation of a high viscosity gel layer around drug contents. Further, this results into decreased mobility of drug in gel matrices, which leads to decrease in release rate. PEG 400 has performed significant role in dissolution. It was found to increase dissolution rate with PEG 400 concentration. Hence, the role of PEG 400 was dissolution facilitating agent in addition to its role as a plasticizer. Comparative study of dissolution of all factorial batches is shown in Figure 1.

The formulation F7 has shown highest dissolution 99.10% within 210 sec in phosphate buffer pH 6.8.

It is well known that amorphous form of drugs produce higher dissolution rates than corresponding crystalline form. In the film formulation, the driving force of crystallisation is reduced due to poor mobility of drug thereby improving physical stability of the amorphous drugs. Polymers are expected to lessen the molecular mobility of the drug within a film.

Film surface became porous forming a network like structure due to presence of HPMC E5 polymer. This porous surface helps in fast disintegration and dissolution by penetrating dissolution medium through pores of network.

**Figure 2:**
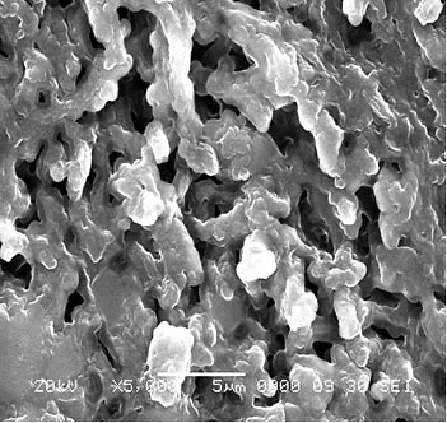
Scanning Electron Micrographs of optimized batch

Surface morphology of film was obtained by SEM images. Scanning electron micrograph of optimized batch is shown in Figure 2 which states that film has a rough, porous surface that helps the medium to get penetrate inside the film.

### Percent drug release

Analysis of variance (ANOVA) of the design model for percent drug release is given. The Model F-value was found 16.14 indicate that 2FI model was significant. As the p-value for design response shows less than 0.05 which signifies that the design model is significant. Equation is,

Percent drug release (Y_1_)** =** 9.611 – 0.129(A) + 0.068(B) – 0.082(A) (B)…..Equation (1)

Where, A = HPMC E5 and B = PEG 400

HPMC E5 (A) shows negative effect and PEG 400 (B) shows positive effect on percent drug release. This is endorsed by elevated level of polymer which results in formation of viscous gel layer due to high dense particles of HPMC E5.

### Disintegration time

The model F- value was found 7.36 indicate that linear model is significant. Values of “Prob>F” less than 0.0500 indicates that model terms are significant.

Equation is,

Disintegration time (Y_2_) = 38.555 + 8.5(A) – (B) - 0.5(A) (B) …..Equation (2)

HPMC E5 shows positive effect on disintegration time. Disintegration time enhances with increase in concentration of HPMC E5. PEG 400 shows negative effect on disintegration time.

### Optimization of factorial batch

Design expert has shown F7 as optimized batch. The batch shows concentration of HPMC E5 and PEG 400 as 300 mg and 0.5mL respectively for formulation. Then, the same batch was further formulated and validated. Results are shown in Table 3.

**Table 3:** Validation of optimization

Sr. no.	Responses	Observed value	Predicted value	Percent error
1	Percent drug release (%)	98.20715	97.8311	+ 0.382
2	Disintegration time (sec)	29.6666	29.5556	+ 0.374

## Conclusion

The challenge of poor solubility of dimenhydrinate was overcome by using ethanol as cosolvent. The films were successfully prepared by using solvent casting method. Disintegration time for optimized batch was 25 sec and drug release in 210 sec (3.5 min), was 99.10 % which were acceptable. Thus, the formulation F7 has potential to develop into a film as solid unit dosage form.

## Acknowledgments

Authors are thankful to Department of Physics, Savitribai Phule Pune University India for providing facility of SEM.

## Ethical Issues

Not applicable

## Conflict of Interest

Authors declare no conflict of interest.
